# The interaction between the measles virus nucleoprotein and the Interferon Regulator Factor 3 relies on a specific cellular environment

**DOI:** 10.1186/1743-422X-6-59

**Published:** 2009-05-15

**Authors:** Matteo Colombo, Jean-Marie Bourhis, Celia Chamontin, Carine Soriano, Stéphanie Villet, Stéphanie Costanzo, Marie Couturier, Valérie Belle, André Fournel, Hervé Darbon, Denis Gerlier, Sonia Longhi

**Affiliations:** 1 Architecture et Fonction des Macromolécules Biologiques, UMR 6098 CNRS et Universités Aix-Marseille I et II, 163 Avenue de Luminy, Case 932, 13288 Marseille Cedex 09, France; 2 Dept of Biomolecular Sciences and Biotechnology, Universita' degli Studi di Milano, Via Celoria, 26. I-20133 Milan, Italy; 3 Institut de Biologie et Chimie des Protéines, UMR 5086 CNRS Université de Lyon, 7, passage du Vercors, 69 367 Lyon cedex 7, France; 4 VirPatH, FRE 3011, CNRS and Université Lyon 1, Faculté de Médecine RTH Laennec, 69372, Lyon, France; 5 Bioénergétique et Ingénierie des Protéines, UPR 9036 CNRS, 31 Chemin Joseph Aiguier, 13402 Marseille Cedex, France, and Université Aix-Marseille I, 3 place Victor Hugo 13331, Marseille, Cedex 3, France

## Abstract

**Background:**

The genome of measles virus consists of a non-segmented single-stranded RNA molecule of negative polarity, which is encapsidated by the viral nucleoprotein (N) within a helical nucleocapsid. The N protein possesses an intrinsically disordered C-terminal domain (aa 401–525, N_TAIL_) that is exposed at the surface of the viral nucleopcapsid. Thanks to its flexible nature, N_TAIL _interacts with several viral and cellular partners. Among these latter, the Interferon Regulator Factor 3 (IRF-3) has been reported to interact with N, with the interaction having been mapped to the regulatory domain of IRF-3 and to N_TAIL_. This interaction was described to lead to the phosphorylation-dependent activation of IRF-3, and to the ensuing activation of the pro-immune cytokine RANTES gene.

**Results:**

After confirming the reciprocal ability of IRF-3 and N to be co-immunoprecipitated in 293T cells, we thoroughly investigated the N_TAIL_-IRF-3 interaction using a recombinant, monomeric form of the regulatory domain of IRF-3. Using a large panel of spectroscopic approaches, including circular dichroism, fluorescence spectroscopy, nuclear magnetic resonance and electron paramagnetic resonance spectroscopy, we failed to detect any direct interaction between IRF-3 and either full-length N or N_TAIL _under conditions where these latter interact with the C-terminal X domain of the viral phosphoprotein. Furthermore, such interaction was neither detected in *E. coli *nor in a yeast two hybrid assay.

**Conclusion:**

Altogether, these data support the requirement for a specific cellular environment, such as that provided by 293T human cells, for the N_TAIL_-IRF-3 interaction to occur. This dependence from a specific cellular context likely reflects the requirement for a human or mammalian cellular co-factor.

## Background

Measles virus (MeV) is an enveloped RNA virus within the *Morbillivirus *genus of the *Paramyxoviridae *family. Its non-segmented, negative-sense, single-stranded RNA genome is encapsidated by the viral nucleoprotein (N) within a helical nucleocapsid. Transcription and replication are carried out onto this N:RNA complex by the viral polymerase complex which consists of two components, the large protein (L) and the phosphoprotein (P) (reviewed in [1]).

MeV N consists of two regions: a structured N-terminal moiety, N_CORE _(aa 1–400), which contains all the regions necessary for self-assembly and RNA-binding [2,3], and a C-terminal domain, N_TAIL_(aa 401–525) that is intrinsically unstructured [4] and is exposed at the surface of the viral nucleocapsid [2,5,6].

Intrinsically disordered proteins (IDPs) or protein domains lack highly populated and uniform secondary and tertiary structure under physiological conditions but fulfill essential biological functions [7-19]. Since N_TAIL _is intrinsically flexible and is exposed at the surface of the viral nucleocapsid, it interacts with various partners, including the viral P protein [3,4] and host cell proteins such as the major inducible heat shock protein (Hsp72) [20,21], and the yet uncharacterized Nucleoprotein Receptor (NR) [22,23]. In addition, it has also been reported to interact with the Interferon Regulator Factor 3 (IRF-3) [24].

**Figure 1:**
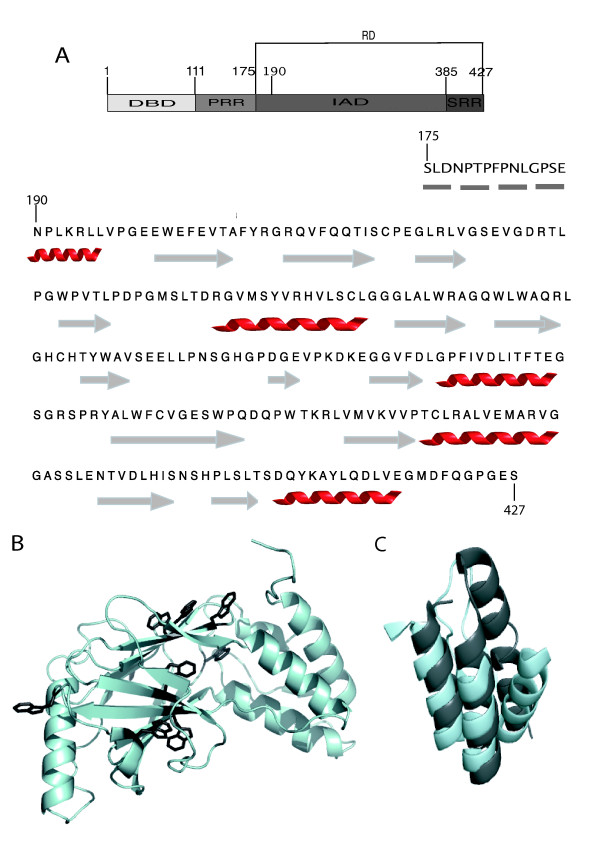
**(A) Schematic representation of the modular organization of IRF-3**. **(B) **Ribbon representation of the crystal structure of IRF-3 RD (pdb code 1QWT) in which the side chains of trp residues are shown in sticks and in dark grey. **(C) **Superimposition between the crystal structure of IRF-3 RD (light grey) and XD (dark grey, pdb code 1OKS).

IRF-3 is ubiquitously expressed as a stable latent transactivator of the cellular innate immune response [25]. It belongs to the family of interferon regulatory factors (IRF) and acts as a transactivator for the interferon-β (IFN-β) and various pro-inflammatory cytokine genes. All mammalian IRFs share a conserved N-terminal DNA binding domain (DBD) and a C-terminal interferon association domain (IAD). IRF-3 consists of a DBD (aa 1–110), of a proline-rich region (PRR, aa 112–174), followed by the IAD (aa 175–384) and by a serine-rich region (SRR, aa 385–427) (Figure 1A).

The seminal and unique observation that MeV N activates IRF-3 to induce CCL5 (also called RANTES), a pro-inflammatory cytokine, but not IFN-β, was done by the Hiscott's group ?[24]. After MeV infection, IRF-3 was phosphorylated at the key Ser^385 ^and Ser^386 ^residues, and this form was able to bind to the interferon sensitive response element of ISG15 in complex with CREB binding protein *in vitro*. Activation of IRF-3, which required active MeV transcription, was also mimicked by the transient expression of the N protein [24]. Moreover, IRF-3 and a cellular kinase could be co-immunoprecipitated with N [24]. From these data it was assumed that MeV N physically interacts with IRF-3 and induces the phosphorylation of the latter by recruiting the kinase. Phosphorylation of IRF-3 would then lead to IRF-3 homo-dimerisation, followed by IRF-3 nuclear import and transactivation of a selective set of pro-inflammatory cytokines [24]. Using deletion constructs and co-immunoprecipitation studies, the IRF-3 binding region was grossly mapped to N_TAIL _(residues 415–523), while the N binding region within IRF-3 was mapped to residues 198–394 [24].

We have previously reported that N_TAIL _undergoes α-helical induced folding upon binding to P [4], and solved the crystal structure of the P domain (XD, aa 459–507) responsible for the N_TAIL _induced folding [26]. Within a conserved region of N_TAIL _(aa 489–506, Box2), we have identified an α-helical molecular recognition element (α-MoRE, aa 489–499) [27], involved in the binding to XD and in induced folding [26,28-30]. XD-induced α-helical folding of the N_TAIL _region encompassing residues 486–503 was confirmed by Kingston and co-workers, who solved the crystal structure of a chimeric construct consisting of XD and the 486–504 region of N_TAIL _[31]. Analysis of this structure revealed that the α-helix of N_TAIL _is embedded in the hydrophobic cleft of XD delimited by helices α2 and α3, to form a pseudo-four helix arrangement that is very frequently found in nature [31].

Analysis of the crystal structure of the regulatory domain (RD, aa 175–427) of IRF-3 (IRF-3 RD, pdb code 1QWT, [32]) (Figure 1B) points out the presence of a triple α-helical bundle well superimposable to the structure of XD (Figure 1C). Furthermore, the triple α-helical bundle of IRF-3 also accommodates the nuclear co-activator binding domain (or IbiD domain) of CREB (pdb code 1ZOQ, [33], data not shown), which forms a disordered molten globule in the absence of a binding partner [34] and that folds into an α-helical structure upon binding to IRF-3 [33]. We therefore hypothesized that the α-helical bundle of IRF-3 may support the ability of IRF-3 to interact with the disordered N_TAIL _domain in a way reminiscent of that of XD.

After confirming the reciprocal ability of IRF-3 and N to be co-immunoprecipitated in human cells, we undertook the cloning and the bacterial expression of IRF-3 RD in view of obtaining conspicuous protein amounts suitable for further biochemical and biophysical studies aimed at investigating the molecular mechanisms of the N_TAIL_-IRF-3 interaction. A monomeric form of IRF-3 RD was then purified from the soluble fraction of *E. coli*, and further used in experiments aimed at ascertaining whether N_TAIL _underwent induced folding upon binding to IRF-3. Using a panel of spectroscopic approaches, including circular dichroism (CD), fluorescence spectroscopy, nuclear magnetic resonance (NMR) and electron paramagnetic resonance (EPR) spectroscopy, we failed to document a direct binding of N_TAIL _with IRF-3 RD, under conditions where the N_TAIL_-XD interaction was detected. Moreover, the lack of direct interaction of IRF-3 with the full-length N protein ruled out a possible contribution of the folded N_CORE _domain of N (aa 1–400) in the interaction with IRF-3. Strikingly, the interaction could not be detected in the bacterial lysate either, nor was it observed using a yeast two hybrid assay. Altogether these results support the requirement of a specific cellular environment for the N_TAIL_-IRF-3 interaction to occur.

## Methods

### Bacterial strains, primers, restriction enzymes and antibodies

The *E. coli *strains DH5α (Stratagene) or Rosetta [DE3] pLysS (Novagen) were used for selection and amplification of DNA constructs, or for the expression of recombinant proteins, respectively.

Primers were from Invitrogen and Operon. Restriction enzymes, anti-flag mAb, and goat anti-mouse HRP conjugated secondary antibodies were purchased from New England Biolabs, Sigma, and Upstate Laboratories, respectively.

### Co-immunoprecipitation of proteins expressed in human cells

The pCDNA-myc-IRF3 and pEF-BOS-flag-N eukaryotic vectors were derived by PCR and subcloning into a home-made pCDNA-myc and pEF-BOS-flagx2 vector so as to encode N-terminal myc- and flag- tagged IRF-3 and N protein, respectively. 293T cells (2 × 10^6^) were cotransfected with 12 μg of plasmid DNA using Dreamfect-Gold reagent according to OZ BIOSCIENCES' instructions . Two days after, cells were collected and lysed in 0.3 ml of lysis buffer (50 mM Tris pH 8.0, 5 mM EDTA, 150 mM NaCl, 0.5% Igepal CA-630 (Sigma), 1 mM phenyl-methyl-sulphonyl-fluoride (PMSF) and 1× Complete^® ^(Roche) by 3 passages into a 26G needle for 30 min on ice. Cell debris were eliminated by centrifugation at 15,000 rpm for 15 min. Then, proteins were immunoprecipitated using rabbit anti-IRF-3 (Santa-Cruz) and protein-G-Sepharose^® ^(GE Healthcare Life Sciences) beads and eluted as detailed elsewhere [35]. Alternatively, they were immunoprecipitated using Monoclonal ANTI-FLAG^® ^M2 Affinity Gel and eluted using 3X FLAG^® ^Peptide according to Sigma's instructions. Proteins were then detected by western blotting using the appropriate antibody and peroxydase-conjugate combinations as detailed elsewhere [35].

### Cloning of human IRF-3 cDNA

The cDNA of human IRF3 was obtained by RT-PCR from total RNA extracted from HeLa cells. The RNA extraction method and the RT-PCR were performed as described elsewhere [36]. The IFR-3 cDNA was PCR amplified using forward 5'-CAT**GAATTC**ATGGGAACCCCAAAGCCA-3' and backward 5'-TGA**CTCGAG**TCAGCTCTCCCCAGGGCC-3' primers containing *EcoR*I and *Xho*I restriction sites (bold), respectively. The cDNA was subcloned downstream the myc tag into an in-house made pcDNA3-myc plasmid. The myc-IRF-3 construct was checked by sequencing.

### Construction of IRF-3 RD expression plasmids

The IRF-3 RD_HN_, IRF-3 RD_FN _and IRF-3 RD_HC _gene constructs, encoding residues 175–427 of the IRF-3 protein with either an hexahistidine tag fused to the N-terminus (IRF-3 RD_HN_) or to the C-terminus (IRF-3 RD_HC_), or with a flag sequence (DYKDDDDK) [37] fused to the N-terminus (IRF-3 RD_FN_), were obtained by recursive PCR, using pIRF-3 as template and *Pfx *polymerase (Invitrogen). Primers were designed to introduce either a hexahistidine tag encoding sequence (either at the N- or at the C-terminus of IRF-3 RD) or a flag encoding sequence at the N-terminus of IRF-3 RD, as well as an *AttB1 *and an *AttB2 *site allowing further cloning into the pDest14 vector (Invitrogen) using the Gateway recombination system (Invitrogen). The sequence of the coding region of all the pDest14/IRF-3 RD constructs was checked by sequencing (GenomeExpress).

### XD, N and N_TAIL _expression plasmids

The following constructs have already been described: *(i) *the pDest14/XD_HC _gene construct, encoding residues 459–507 of the MeV P protein (strain Edmonston B) with a hexahistidine tag fused to its C-terminus, [26], *(ii) *the N gene construct, pet21a/N_FNHC_, encoding the MeV N protein (strain Edmonston B) with a flag fused at its N-terminus and an hexahistidine tag fused to its C-terminus, [2], *(iii) *the pDest14/N_TAILHN_, encoding residues 401–525 of the *wt *MeV N protein (strain Edmonston B) with an N-terminal hexahistidine tag [38], and *(iv) *the N_TAIL _S407C, N_TAIL _L496C and N_TAIL _V517C gene constructs, encoding residues 401–525 of the MeV N protein with a Cys substitution at positions 407, 496 and 517 of N, respectively, and with a N-terminal hexahistidine tag [29].

The pDest14/N_TAILFN _construct, encoding residues 401–525 of the *wt *MeV N protein (strain Edmonston B) with an N-terminal flag sequence (N_TAILFN_), was obtained by recursive PCR followed by cloning into the pDest14 vector. PCR was carried out using pDest14/N_TAILHN _[38] as template, and *Pfu *polymerase (Promega). Beyond *AttB1 *and *AttB2 *sites, primers were designed to introduce a flag encoding sequence at the N-terminus of N_TAIL_. The coding regions of the N_TAILFN _construct was checked by sequencing (GenomeExpress).

### Expression of recombinant proteins

The *E. coli *strain Rosetta [DE3] (Novagen) was used for the expression of the pDest14/IRF-3 RD constructs. Cultures were grown overnight to saturation in Luria-Bertani medium containing 100 μg/ml ampicilin and 17 μg/ml chloramphenicol. An aliquot of the overnight culture was diluted 1/12.5 in LB medium and grown at 37°C. At OD_600 _of 0.7, the culture was incubated in ice for 2 hours. Then isopropyl β-D-thiogalactopyranoside (IPTG) and ethanol were added to a final concentration of 50 μM and 2% (v/v), respectively. Cells were grown at 17°C for 16 hours. The induced cells were harvested, washed and collected by centrifugation. The resulting pellets were frozen at -20°C.

Isotopically substituted (^15^N) N_TAIL _and (^15^N) IRF-3 RD were prepared by growing bacteria transformed by the pDest14/N_TAILHN _and pDest14/IRF-3RD_HN _constructs, respectively, in minimal M9 medium supplemented with ^15^NH_4_Cl (0.8 g/l) [38]. Expression of tagged XD (XD_HC_) [26], tagged N [2],*wt *and cys-substituted N_TAIL _[4,29,38] proteins was carried out as already described.

### Purification of recombinant proteins

Cellular pellets from bacteria transformed with the pDest14/IRF3-RD_HN _expression plasmid were resuspended in 5 volumes (v/w) buffer A (50 mM sodium phosphate pH 8, 300 mM NaCl, 10 mM Imidazole, 1 mM PMSF supplemented with lysozyme 0.1 mg/ml, DNAse I 10 μg/ml, protease inhibitor cocktail (Complete ^® ^Roche) (one tablet per 50 ml of lysis buffer). After a 20 min incubation with gentle agitation, the cells were disrupted by sonication (using a 750 W sonicator and 4 cycles of 30 s each at 60% power output). The lysate was clarified by centrifugation at 30,000 g for 30 min. Starting from one liter of culture, the clarified supernatant was incubated for 1 h with 4 ml Talon resin (Clontech), previously equilibrated in buffer A. The resin was washed with buffer A, and the IRF-3 RD protein was eluted in buffer A containing 250 mM imidazole. Eluates were analyzed by SDS-PAGE for the presence of the desired product. The fractions containing the recombinant product were combined, dialyzed against buffer B (20 mM Tris/HCl pH 7.4, 10 mM NaCl, 0.1 mM EDTA, 1 mM DTT) and then loaded onto a Hi-Trap Q Fast-Flow 5 × 1 column (GE Healthcare). The protein was eluted with a NaCl gradient (from 25 to 250 mM). The fractions containing the protein were combined and concentrated using 10 kDa molecular cutoff Centricon Plus-20 (Millipore) prior to loading onto a Superdex 200 HR 10/30 column (GE Healthcare) followed by elution in various buffers. After elution with buffer C (20 mM Hepes pH 7.3, NaCl 100 mM, EDTA 0.1 mM), the fractions corresponding to IRF3 were collected and dialyzed against buffer D (20 mM Hepes pH 7.3, NaCl 10 mM, EDTA 0.1 mM). The purified protein, referred to as IRF-3 RD, was stored at -20°C.

Purification of histidine-tagged N, XD and of *wt *or cys-substituted N_TAIL _proteins was carried out as described in [2,26,29,30,38].

All purification steps, except for gel filtrations, were carried out at 4°C. Protein concentrations were calculated using OD_280 _measurements and the theoretical absorption coefficients ε (mg/ml.cm) at 280 nm as obtained using the program ProtParam at the EXPASY server . Apparent molecular mass of proteins eluted from gel filtration columns was deduced from a calibration carried out with Low Molecular Weight (LMW) and High Molecular Weight (HMW) calibration kits (GE Healthcare). The theoretical Stokes radius (R_s_) of a native (R_s_N) protein was calculated according to [39]: log(R_s_N) = 0.369*log(MM) - 0.254, with (MM) being the molecular mass (in Daltons) and R_S _being expressed in Å.

### Analytical size-exclusion chromatography (SEC) with on-line multi-angle laser light-scattering, absorbance, and refractive index (MALS/UV/RI) detectors

SEC was carried out on a HPLC system (Alliance 2695, Waters) using a Superose 12 column (5 ml) (Amersham, Pharmacia Biotech) eluted with various buffers at a flow of 0.5 ml/min. Detection was performed using a triple-angle light-scattering detector (MiniDAWN™ TREOS, Wyatt Technology), a quasi-elastic light-scattering instrument (Dynapro™, Wyatt Technology) and a differential refractometer (Optilab^® ^rEX, Wyatt Technology). Molecular mass and hydrodynamic radius (Stokes radius, R_S_) determination was performed by the ASTRA V software (Wyatt Technology) using a *dn/dc *value of 0.185 ml/g. IRF-3 RD was loaded at a final concentration ranging from 0.2 mM to 1.2 mM.

### Dynamic Light Scattering (DLS)

Dynamic light scattering experiments were performed with a Nano-S Zetasizer (MALVERN) at 20°C. All samples were filtered prior to the measurements (Millex syringe filters 0.22 μm, Millipore). The hydrodynamic radius was deduced from translational diffusion coefficients using the Stokes-Einstein equation. Diffusion coefficients were inferred from the analysis of the decay of the scattered intensity autocorrelation function. All calculations were performed using the software provided by the manufacturer.

### Mass Spectrometry (MALDI-TOF)

Mass analysis of tryptic fragments was carried out using an Autoflex mass spectrometer (Bruker Daltonics). 1 μg of purified IRF-3 RD obtained after separation onto 12% SDS-PAGE was digested with 0.25 μg trypsin. The experimental mass values of the tryptic fragments were compared to theoretical values found in protein data base . The mass standards were either autolytic tryptic peptides or peptide standards (Bruker Daltonics).

### Spin labeling and EPR spectroscopy

Spin labeling of cysteine-substituted N_TAIL _variants was carried out as already described [29,30]. EPR spectra were recorded at room temperature on an ESP 300E Bruker spectrometer equipped with an ELEXSYS Super High Sensitivity resonator operating at 9.9 GHz. Samples were injected in a quartz capillary, whose sensitive volume was about 20 μl. The microwave power was 10 mW and the magnetic field modulation frequency and amplitude were 100 kHz and 0.1 mT, respectively. Spectra were recorded in buffer D. The concentration of spin-labeled N_TAIL _variants was 20 μM, while that of IRF-3 RD was 80 μM.

### Circular Dichroism

Circular dichroism (CD) spectra were recorded on a Jasco 810 dichrograph using 1 mm thick quartz cells at 20°C. All spectra were recorded in 10 mM sodium phosphate buffer pH 7.0.

CD spectra were measured between 185 and 260 nm, at 0.2 nm/min and were averaged from three independent acquisitions. The spectra were corrected for water signal and smoothed by using a third-order least square polynomial fit. Protein concentrations of 0.1 mg/ml were used. Mean ellipticity values per residue ([Θ]) were calculated as [Θ] = 3300 m ΔA/(l c n), where l (path length) = 0.1 cm, n = number of residues, m = molecular mass in daltons and c = protein concentration expressed in mg/ml.

Structural variations of N_TAIL _upon addition of IRF-3 RD were measured as a function of changes in the initial CD spectrum upon addition of two-fold molar excess of IRF-3 RD. XD was used as a positive control.

The number of residues (n) is 132 for N_TAILHN_, 260 for IRF-3 RD, and 56 for XD, while m values are 14,632 Da for N_TAILHN_, 28,903 Da for IRF-3 RD, and 6, 686 Da for XD. In the case of protein mixtures, mean ellipticity values per residue ([Θ]) were calculated as [Θ] = 3300 ΔA/{[(C_1 _n_1_)/m_1_) + (C_2 _n_2_/m_2_)] l}, where l (path length) = 0.1 cm, n_1 _or n_2 _= number of residues, m_1 _or m_2 _= molecular mass in daltons and c_1 _or c_2 _= protein concentration expressed in mg/ml for each of the two proteins in the mixture. The average ellipticity values per residue ([Θ]_Ave_), were calculated as follows: [Θ]_Ave _= [([Θ]_1 _n_1_) + ([Θ]_2 _n_2_R)]/(n_1 _+ n_2 _R), where [Θ]_1 _and [Θ]_2 _correspond to the measured mean ellipticity values per residue, n_1 _and n_2 _to the number of residues for each of the two proteins, and R to the excess molar ratio of protein 2. The experimental data in the 185–260 nm range were treated using the CDNN software package, which allowed estimation of the α-helical content.

### Fluorescence spectroscopy

Fluorescence intensity variations of IRF-3 RD tryptophans were measured by using a Cary Eclipse (Varian) equipped with a front-face fluorescence accessory at 20°C, by using 2.5 nm excitation and 10 nm emission bandwidths. The excitation wavelength was 290 nm and the emission spectra were recorded between 300 and 450 nm. Titrations were performed in a 1 ml quartz fluorescence cuvette containing 1 μM IRF-3 RD in buffer D, and by gradually increasing the concentration of N_TAIL _from 10 nM to 1 μM. Experimental fluorescence intensities were corrected by subtracting the spectrum obtained with N_TAIL _protein alone (note that N_TAIL _is devoid of tryptophan residues). Data were analyzed by plotting the relative fluorescence intensities at the maximum of emission at increasing N_TAIL _concentrations.

### Two-dimensional Heteronuclear Magnetic Resonance

2D-HSQC spectra [40] were recorded on a 600-MHz ultra-shielded-plus Avance-III Bruker spectrometer equipped with a TCI cryo-probe. The temperature was set to 300 K and the spectra were recorded with 2048 complex points in the directly acquired dimension and 128 points in the indirectly detected dimension, for 6 h each. Solvent suppression was achieved by the WATERGATE 3–9–19 pulse [41]. The data were processed using the TOPSPIN software, and were multiplied by a sine-squared bell and zero-filled to 1k in first dimension with linear prediction prior to Fourier transform.

The samples were *(i) *a 25 μM uniformly ^15^N-labeled N_TAILHN _either alone or after addition of a 4-fold molar excess of IFR-3 RD, and *(ii) *a 25 μM uniformly ^15^N-labeled IRF-3 RD either alone or after addition of a 2-fold molar excess of full-length N. Spectra were recorded in buffer D containing 10% D_2_O (v/v).

### Co-immunoprecipitation of proteins expressed in bacteria

Twenty to 80 ml aliquots of induced bacterial cultures expressing either N_TAIL _or IRF-3 RD, were harvested, washed, collected by centrifugation and the resulting pellets were frozen at -20°C. Aliquots were individually resuspended in 500 μl of buffer C supplemented with 1 mM PMSF, 0.1 mg/ml lysozyme, 10 μg/ml DNAse I, and protease inhibitor cocktail (Complete ^® ^Roche). Bacterial lysates were sonicated (using a 750 W sonicator and 3 cycles of 7 s at 35% power output) and were clarified by centrifugation at 16,000 g for 20 min at 4°C. The supernatants, were recovered and filtered onto 0.45 μm Ultrafree-MC centrifugal filter devices (Millipore).

Fifty to 100 μl of a bacterial lysate expressing a flagged protein (N_TAIL _or IRF-3 RD, lysate A) were mixed with 60 μg of an anti-flag monoclonal antibody (Sigma-Aldrich), 15 μl of Protein A-Sepharose CL 4B (GE Healthcare) (previously equilibrated with 10 volumes of buffer C), and buffer C up to a final volume of 400 μl to increase the volume during the binding step. After 1 h incubation at 4°C with gentle agitation, the flow-through was recovered and the resin was washed twice with 20 bed volumes of buffer C. Fifty μl of either a bacterial lysate expressing an unflagged protein (N_TAIL_, lysate B) or of buffer C containing 5 μg of purified unflagged XD (protein B), both corresponding to stoichiometric amounts, were added to the resin and incubation was carried out for one additional hour. The flow-through, containing the unretained fraction, and the resin were recovered and analyzed by SDS-PAGE. The N_TAIL_-XD couple was used as the positive control. Additional controls included incubation of the immobilized immunoaffinity chromatography (IIAC) resin with either lysates A or lysates/proteins B alone (data not shown). The identity of the co-precipitated or unretained protein bands was confirmed by mass-spectrometry.

### Yeast two-hybrid assay

The following constructs were made by PCR amplification using the pGBKT7-N_TAIL _plasmid [42] as a template: MeV N_TAIL _(N 401–525), N_TAIL_Δ1 (N 421–525), N_TAIL_Δ2,3 (N 401–488), N_TAIL_Δ3 (N 401–516). They were cloned in-frame downstream the GAL4 DNA-binding domain of the pLexAGagB vector (Aptanomics) thus yielding BD-bait fusion proteins named BD-N_TAIL_, BD-N_TAIL_Δ1, BD-N_TAIL_Δ2,3, BD-N_TAIL_Δ3. PCT (P 231–507) from pGBKT7-PCT plasmid [42] and IRF-3 from pcDNA3-myc-IRF-3 were cloned in-frame downstream the GAL4-activating domain of the vector pWP2C (Aptanomics) to yield the AD-PCT and AD-IRF3 proteins, respectively. The pLexA (no protein in fusion, Ø), pWP2::RG22C anti-LexA (Ctr+) and pWP2::C5C (Ctr-) plasmids (Aptanomics) were used as controls. All plasmids were checked by sequencing. MB226α (Leu-Trp-His-Ade-) yeast cells transformed with the BD-bait and pSH1834 (coding for β-galactosidase as reporter gene) vectors, and MB210a (MATα, Leu-Trp-His-Ade-) yeast cells transformed with the AD-prey vectors, were selected on histidine + uracile (SD/-His-Ura), and tryptophan (SD/-Trp) deficient SD medium, respectively. Transformed MB226α and MB210a cells were mated and grown on Glucose -His-Ura-Trp+X-Gal for successful mating with replicate on Galactose/Raffinose -His-Ura-Trp+X-Gal for testing the interaction between baits and preys. Experiments were repeated two times. Expression of bait and prey fusion were verified by western blot using anti-HA monoclonal antibody as described previously [42].

## Results

### Reciprocal coimmunoprecipitation of myc-IRF-3 and flag-N proteins

**Figure 2:**
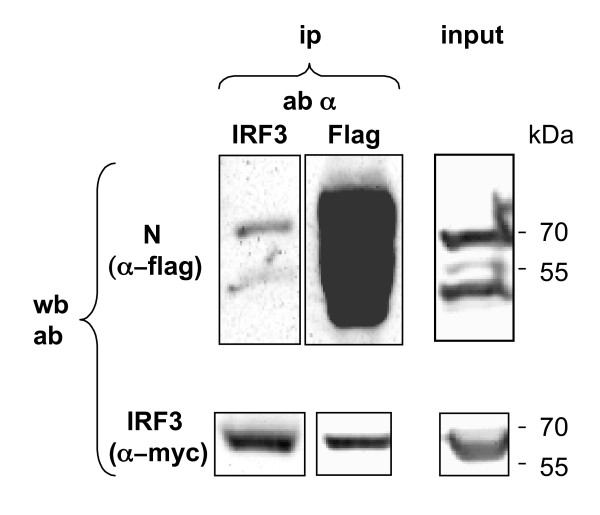
**Reciprocal coimmunoprecipitation of myc-IRF-3 and flag-N proteins**. Flag-N was co-expressed with myc-IRF3 in 293T cells and immunoprecipitated by either anti-flag mAb or rabbit polyclonal anti-IRF3 antibodies. After elution by Flagx3 peptide or Laemli buffer, immunoprecipitated proteins were analysed by Western Blotting using either anti-flag or anti-myc mAb. Note that the western blot was overexposed so as to reveal flag-N co-immunoprecipitated with myc-IRF-3

When co-expressed in human 293T cells, flag-N and myc-IRF3 formed complexes that could be co-immunoprecipitated by either anti-Flag or anti-IRF-3 antibodies (Figure 2). However, the amount of N found in the anti-IRF-3 immunoprecipitate was rather limited, since it was detected only after overexposure of the western blot, a condition where the N signal immunoprecipitated by anti-Flag antibodies is very intense. As controls N and P proteins were readily co-immunoprecipitated, while no myc-IRF3/P complex was detected, thus ruling out the possibility that IRF-3 might be aspecifically retained onto the resin (data not shown). Cells expressing myc-IRF3 were used to ascertain antibody specificity in the western blot assay. These results thus confirm those previously obtained by ten Oever *et al *[24]

### Domain analysis of IRF-3 and subcloning of the IRF-3 gene fragment encoding the regulatory domain (RD)

IRF-3 has a modular organization (see Figure 1A), with the N_TAIL _binding region having been mapped to residues 198–384 [24]. Since the IRF-3 region encompassing residues 175–427 (herein referred to as regulatory domain, RD) was successfully purified from the soluble fraction of *E. coli *and further crystallized [32], we cloned the DNA fragment of the IRF-3 gene encoding RD into the pDest14 expression plasmid. The resulting constructs encode RD with either an N-terminal or a C-terminal histidine tag.

### Expression and purification of a stable, monomeric form of IRF-3 RD

**Figure 3:**
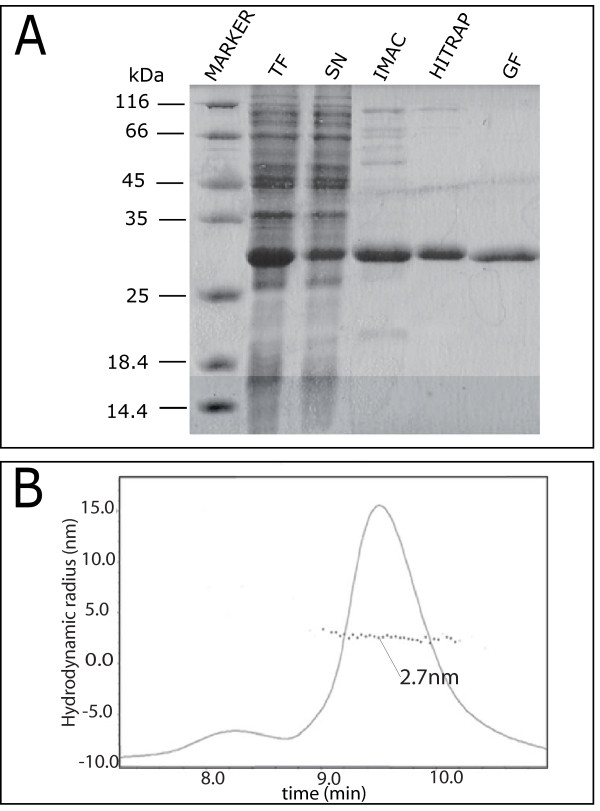
**Purification of IRF-3 RD from *E. coli***. **(A) **Coomassie blue staining of a 15% SDS-PAGE. TF: bacterial lysate (total fraction); SN: clarified supernatant (soluble fraction); IMAC: eluent from Immobilized Metal Affinity Chromatography; HITRAP: eluent from Ion Exchange Chromatography. GF: eluent from Gel Filtration. **(B) **Elution profile of IRF-3 RD from analytical SEC-MALS in buffer C. The peak containing IRF-3 RD is highlighted and the inferred R_S _is also shown.

While the construct encoding IRF-3 RD with a histidine-tag at the C-terminus was poorly expressed and mostly insoluble upon induction at 17°C (data not shown), the construct bearing the histidine-tag at the N-terminus was well expressed and its solubility was estimated to be approximately 50% (Figure 3A). IRF-3 RD was purified to homogeneity (> 95%) in 3 steps: immobilized metal affinity chromatography (IMAC), ion exchange chromatography (IEC) and gel filtration (Figure 3A). The identity of the recombinant product was confirmed by mass spectrometry analysis of the tryptic fragments obtained after digestion of purified IRF-3 RD.

IRF-3 was reported to undergo dimerization upon phosphorylation induced by MeV N [24,32]. We indeed found that purified, recombinant IRF-3 RD is a dimer under various buffer conditions, including 10 mM sodium phosphate pH 7 or buffer A (data not shown). Since IRF-3 dimerization is the result of a cascade of events triggered by the initial binding of N, we reasoned that the dimeric form of IRF-3 might in principle be expected to exhibit a reduced ability to bind to N. In support of this hypothesis, heteronuclear NMR, EPR and fluorescence experiments carried out with a dimeric form of IRF-3 RD, showed no detectable interaction with N_TAIL _(data not shown).

We therefore screened various combinations of buffers, ionic strengths and salt concentrations in order to identify conditions where IRF-3 RD is a stable monomer. We used SEC-MALS to assess the oligomeric state of purified IRF-3 RD in various buffers. The experimentally observed R_S _of IRF-3 RD at 0.2 mM in 20 mM Hepes pH 7.3, NaCl 100 mM, EDTA 0.1 mM (buffer C) was 2.7 nm (Figure 3B), which corresponds to the theoretical value expected for a monomer (approximately 2.5 nm) [39]. Moreover, the sharpness and symmetry of the peak indicates the presence of a well-defined molecular species, thus pointing out the homogeneity of the protein sample. Notably, in these buffer conditions, the protein was found to be monomeric in the 0.2–1.2 mM concentration range, thus ruling out a possible effect of sample concentration on oligomerization. DLS analysis showed that the protein remains monomeric in the 0.2–1.2 mM range also after lowering the salt concentration to 10 mM (buffer D). Stability and homogeneity of the sample in buffer D upon prolonged storage at -20°C were checked by DLS. As the oligomeric state of IRF-3 RD was affected by pH and buffer, all subsequent studies, with the only exception of CD experiments, were carried out in buffer D.

### Analysis of the N_TAIL_-IRF-3 RD interaction by circular dichroism

**Figure 4:**
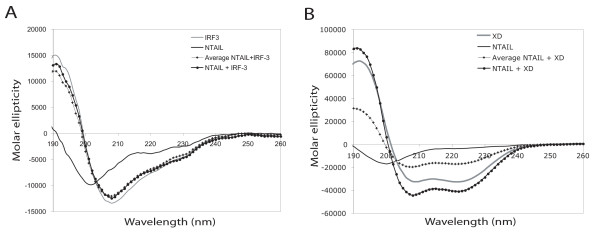
**Analysis of N_TAIL _structural transitions in the presence of IRF-3 RD or XD by far-UV CD**. Far-UV CD spectra of N_TAIL _alone (black line) or in the presence of a two-fold molar excess of IRF-3 RD **(A) **or XD **(B)**. Spectra were recorded in 10 mM sodium phosphate buffer at pH 7. In the mixture containing N_TAIL _+ IRF-3 RD, the concentration of N_TAIL _is 1.4 μM, while that of IRF-3 RD is 2.8 μM. In the mixture containing N_TAIL _+ XD, the concentration of N_TAIL _is 3.5 μM, while that of XD is 7 μM. The CD spectra of XD or IRF-3 RD alone (grey lines), as well as the theoretical average curves calculated by assuming that no structural variations occur (see Materials and Methods) are also shown. Data are representative of one out of three independent experiments.

To ascertain that the purified IRF-3 RD protein was properly folded, we recorded its far-UV CD spectrum. Because of significant absorption of buffer D resulting in highly noisy spectra, the protein (200 μM in buffer D) was diluted to a final concentration of 0.1 mg/ml (3.5 μM) in 10 mM sodium phosphate buffer pH 7. Since the protein was diluted more than 50 times, dimerization under these conditions was assumed to be unlikely. The far-UV CD spectrum of IRF-3 RD (Figure 4A, grey line) is typical of a structured protein with a predominant α-helical content, as indicated by the positive ellipticity between 185 and 200 nm, and by the two minima at 208 and 222 nm. The calculated helicity (28.5%), as obtained using the CDNN software, is in agreement with the α-helical content (26.1%) derived from the analysis of the crystal structure of IRF-3 RD (pdb code 1QWT), thus indicating that the recombinant IRF-3 RD protein is properly folded.

We next addressed the question as to whether IRF-3 RD is able to induce α-helical folding of N_TAIL_, as already reported for XD [26]. We therefore, recorded the far-UV CD spectrum of N_TAIL _in the presence of a two-fold molar excess of IRF-3 RD (Figure 4A), a condition where XD induces α-helical folding of N_TAIL _(Figure 4B). The far-UV CD spectrum of XD (Figure 4B, grey line) is typical of a structured protein with a predominant α-helical content. After mixing N_TAIL _with a two-fold molar excess of XD, the observed CD spectrum differs from the corresponding average curve calculated from the two individual spectra (Figure 4B). Since the average curve corresponds to the spectrum that would be expected if no structural variations occur, deviations from this curve indicate structural transitions. The observed deviations are consistent with an XD-induced α-helical transition of N_TAIL_, as judged by the much more pronounced minima at 208 and 222 nm, and by the higher ellipticity at 190 nm of the experimentally observed spectrum compared to the corresponding average curve (Figure 4B) [26]. Contrary to XD, the experimentally observed CD spectrum of a mixture containing N_TAIL _and a two-fold molar excess of IRF-3 RD very well superimposes onto the average spectrum, thus indicating that N_TAIL _undergoes little, if any, structural transitions in the presence of IRF-3 RD (Figure 4A). A further increase in the molar excess of IRF-3 RD resulted in strong dampening of the N_TAIL _signal due to the larger protein size of IRF-3 RD (28 kDa) as compared to N_TAIL _(14.6 kDa) (data not shown). Increasing the N_TAIL _molar excess did not result in any detectable structural transitions either (data not shown).

### Analysis of the N_TAIL_-IRF-3 RD interaction by heteronuclear NMR spectroscopy

**Figure 5:**
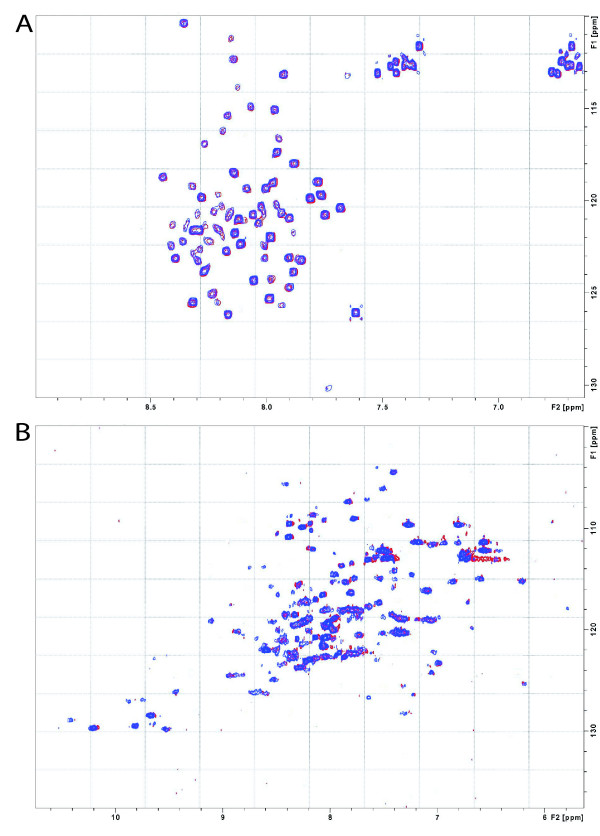
**Analysis of N_TAIL _and IRF3 mutual structural transitions by heteronuclear NMR**. 2D-HSQC of ^15^N-N_TAIL _alone (25 μM) (in blue) or in the presence of unlabelled IRF-3 (100 μM) (in red) **(A) **and of ^15^N-IRF-3 alone (25 μM) (in blue) or in the presence of unlabelled N (50 μM) (in red) **(B)**. Spectra were recorded in buffer D.

We next carried out heteronuclear NMR experiments which allowed the use of buffer D, a condition where IRF-3 RD is monomeric, as well as higher concentrations (100 μM) of the protein partner. The HSQC spectrum of ^15^N uniformly labeled N_TAIL _either alone (25 μM) or in the presence of a four-fold molar excess of unlabeled IRF-3 RD was recorded. The very low spread of the resonance frequencies of N_TAIL _was typical of a disordered protein devoid of stable, highly populated secondary structure (Figure 5A) (see also [4,38]). The HSQC spectrum obtained in the presence of a molar excess of unlabeled IRF-3 RD, pretty well superimposes onto that of N_TAIL _alone, thus pointing out that the ^15^N and ^1^H_N _resonance frequencies of N_TAIL _were not affected by the addition of IRF-3 RD. These data clearly indicate a lack of interaction between N_TAIL _and IRF-3 RD.

In order to assess whether N_TAIL _was able to interact with IRF-3 RD only in the context of the full-length, auto-assembled nucleoprotein, a HSQC spectrum of ^15^N uniformly labeled IRF-3 RD, either alone (25 μM) or in presence of a 2-fold molar excess of full-length nucleoprotein was also recorded. The HSQC spectrum of ^15^N IRF-3 RD was typical of a folded protein, as judged on the basis of the spread of the proton resonances in the 7 to 10 ppm range (Figure 5B). Notably, after addition of unlabeled N, no peak displacement was observed (Figure 5B).

In conclusion, upon mixing IRF-3 RD with either N_TAIL _or the full-length nucleoprotein no magnetic perturbation of the labeled protein was observed.

### Analysis of the N_TAIL_- IRF-3 RD interaction by site-directed spin-labeling EPR spectroscopy

The ability of N_TAIL _to interact with IRF-3 RD was next assessed by using site-directed spin-labeling (SDSL) EPR spectroscopy. The basic strategy of this technique involves the introduction of a paramagnetic nitroxide side chain at a selected protein site. This is usually accomplished by cysteine-substitution mutagenesis, followed by covalent modification of the unique sulfydryl group with a selective nitroxide reagent, such as the methanethiosulfonate (MTSL) derivative (for reviews see [43-45]). Then, EPR spectroscopy is used to monitor variations in the mobility of the spin label in the presence of ligands or protein partners.

**Figure 6:**
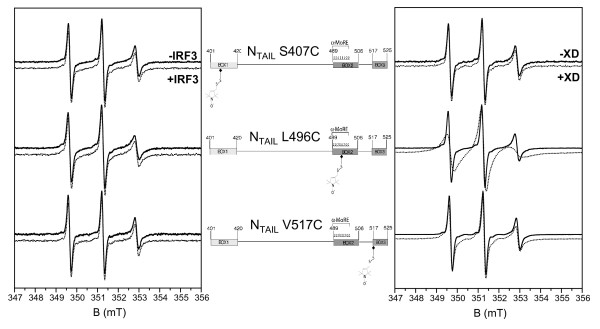
**Analysis of N_TAIL _structural transitions in the presence of IRF-3 RD by EPR spectroscopy**. Normalized room temperature EPR spectra of three spin-labeled N_TAIL _proteins (20 μM) either in the absence or presence of a four-fold molar excess of IRF-3 RD (left panel) or in the absence or presence of a two-fold molar excess of XD (right panel). Spectra were recorded in buffer D. The schematic representation of each spin-labeled N_TAIL _protein is shown. The spin-label is highlighted.

We thus used three spin-labeled N_TAIL _variants, namely S407C, L496C and V517C, which possess a nitroxide spin label covalently grafted at positions 407, 496 and 517, respectively (Figure 6) [29]. We then recorded the EPR spectra of these spin-labeled N_TAIL _proteins either alone (Figure 6, solid line) or in the presence of either a four-fold molar excess of IRF-3 RD (Figure 6, left panel, dotted line) or of a two-fold molar excess of XD (Figure 6, right panel, dotted line). Experiments were carried out in buffer D, a condition where IRF-3 RD is monomeric. The addition of a two-fold molar excess of XD significantly affects the spectral shape of the spin-labeled L496C and V517C N_TAIL _variants, with strong and moderate effects, respectively (Figure 6, right panel) (see also [29]). Conversely, no significant impact of XD on the mobility of the spin label grafted at position 407 was observed (Figure 6, right panel) (see also [29]), in agreement with the well-established lack of involvement of this site in binding to XD [38]. Notably, addition of IRF-3 RD does not trigger any significant variation in the spectral shape in any of the spin-labeled N_TAIL _variants (Figure 6, left panel), thus supporting lack of involvement of the N_TAIL _regions close to the spin-label in the interaction with IRF-3 RD.

### Analysis of the N_TAIL_- IRF-3 RD interaction by intrinsic fluorescence spectroscopy

We next studied the possible impact of N_TAIL _on the fluorescence emission of IRF-3 RD. While N_TAIL _is devoid of trp, IRF-3 RD possesses 9 trp residues. IRF-3 RD has a maximum of fluorescence emission at 343 nm (data not shown). Titration experiments were performed in buffer D, a condition where IRF-3 RD is monomeric. Addition of gradually increasing N_TAIL _concentrations (from 1 nM up to 1 μM) did not trigger any significant variation in the wavelength of emission or in the fluorescence intensity of IRF-3 RD (data not shown), indicating that the chemical environment of the trp residues of IRF-3 RD is not modified in the presence of N_TAIL_.

### Analysis of the N_TAIL_-IRF-3 RD interaction in bacterial lysates

In order to assess whether the interaction between N_TAIL _and IRF-3 RD required a cellular co-factor possibly occurring in prokaryotic cells, we tested the N_TAIL_-IRF-3 RD interaction in bacterial lysates by using co-immunoprecipitation. All experiments were carried out in buffer C, thus ensuring a monomeric state of IRF-3 RD.

**Figure 7:**
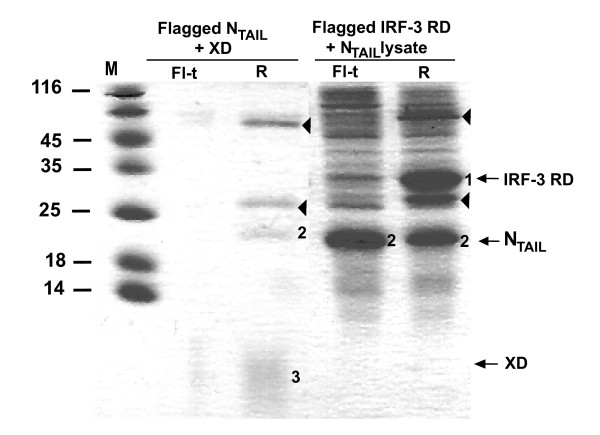
**Co-immunoprecipitation by an anti-flag mAb**. Coomassie blue staining of a 18% SDS-PAGE. Bacterial lysates expressing flagged N_TAIL_, flagged IRF-3 RD, or histidine-tagged N_TAIL _were used. Purified unflagged XD was also used. Fl-t: flow-through (unretained fraction). R: retained fraction. Arrowheads show the light and heavy chains of the mAb, which are visible on the gel at around 25 and 55 kDa, respectively. Numbers 1 to 3 highlight IRF-3 RD, N_TAIL _and XD bands, respectively.

After incubating stoichiometric amounts of histidine tagged XD with a resin coated with an anti-flag mAb and flagged N_TAIL_, XD was only found in the retained fraction, consistent with the ability of these proteins to interact (Figure 7). Conversely, upon addition of stoichiometric amounts of a bacterial lysate expressing histidine tagged N_TAIL _to a resin coated with IRF-3 RD, N_TAIL _was found in both unretained and retained fractions (Figure 7). Note that the occurrence of unflagged N_TAIL _in the retained fraction was not due to the ability of IRF-3 RD to co-precipitate the former on the resin, but rather to the lack of a washing step, thus leading to an equal repartition of N_TAIL _in the unretained and retained fractions. These results showed that while the monoclonal anti-flag antibodies co-immunoprecipitated N_TAIL _and purified XD, they failed to co-immunoprecipitate IRF-3 RD and N_TAIL _from bacterial lysates.

### Analysis of interaction in yeast using the two-hybrid assay

**Figure 8:**
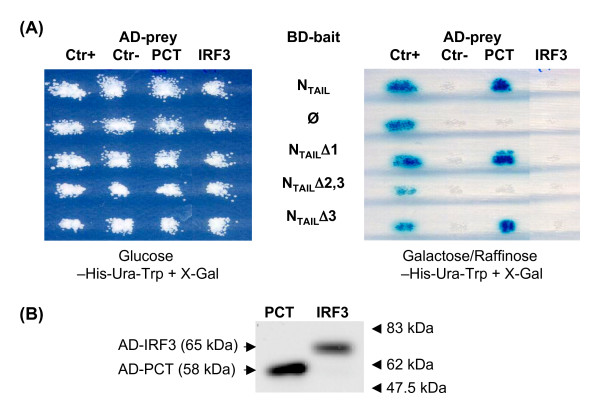
**Analysis of IRF-3 and N_TAIL _interaction in yeast**. **(A) **Yeast growth and X-gal expression after co-transformation with bait-LexA and BD-prey encoding plasmid in glucose -His-Trp + X-Gal and in galactose/raffinose -His-Ura-Trp + X-Gal medium. **(B) **IRF-3-LexA and PCT-LexA expression level in yeast detected by western blot using anti-HA mAb. Ctr+ is the anti-LexA peptide aptamer RG22C, Ctr- is the irrelevant peptide aptamer C5C. Ø indicates LexA alone.

To assess whether the interaction between IRF-3 and MeV N_TAIL _could require an eukaryotic cellular context, we studied this interaction in yeast using the Lex-A two hybrid assay. The successful mating of yeast cells expressing AD-prey and BD-bait constructs was verified by the growth in the glucose-His-Trp + X-Gal supplemented medium (Figure 8A, left panel). Despite the expression of the protein (Figure 8B), the full length IRF-3 fused to Lex-A-activating domain (AD-IRF-3) did not react with any of the BD-N_TAIL _constructs (Figure 8A) as shown by lack of growth in galactose/raffinose -His-Ura-Trp + X-Gal medium and lack of the reporter β-galactosidase enzymatic activity. As controls, an AD-peptide aptamer anti-LexA (Ctr+), but not an irrelevant AD-aptamer (Ctr-), reacted with all Lex-A-baits, while AD-PCT reacted only with BD-N_TAIL_, BD-N_Δ1 _and BD-N_ΔN3_, and not with BD-Ø or with BD-N_Δ2,3 _as expected.

## Discussion

We herein report the bacterial purification of the regulatory domain of IRF-3 and showed that the buffer conditions strongly affect its oligomerization state. Indeed, the protein was shown to exist in either a dimeric or monomeric state depending on the buffer and the ionic strength. Since MeV N was reported to trigger the phosphorylation-dependent dimerization of IRF-3 [24,32], we reasoned that the dimeric form of this latter might in principle be expected to exhibit a reduced or null ability to interact with N_TAIL_. This hypothesis was indeed experimentally confirmed, where various spectroscopic approaches failed to detect an interaction between N_TAIL _and the dimeric form of IRF-3 RD (data not shown). We therefore searched for conditions where IRF-3 RD was found to be a stable monomer up to protein concentrations as high as 1 mM. We then used the monomeric form of IRF-3 RD for a thorough analysis of its ability to interact with N_TAIL_. Using various spectroscopic approaches, we failed to point out any detectable interaction between IRF-3 RD and N_TAIL _under conditions where this latter interacts with the X domain of the phosphoprotein

Lack of deviations of the experimentally observed CD spectrum of an N_TAIL_/IRF-3 RD mixture from the average CD spectrum can be accounted for by assuming that either N_TAIL _does not interact with IRF-3 RD under these experimental conditions, or that their interaction does not imply any significant, concomitant structural rearrangement. It should be pointed out that this spectroscopic approach has been already shown to be sensitive enough to unveil α-helical transitions involving as few as 17 N_TAIL _residues out of 125 [26,28] (see also Figure 3B). That CD was sensitive enough to detect a possible N_TAIL _folding of the same extent as that observed in the presence of XD, was checked and confirmed by the fact that the experimentally observed CD spectrum of a 1:2 mixture of N_TAIL _and IRF-3 RD significantly deviates from a simulated CD spectrum corresponding to a 1:2 mixture of "folded" N_TAIL _and IRF-3 RD. The CD spectrum of "folded" N_TAIL _was calculated from the CD spectrum of a mixture containing N_TAIL _and XD in the 1:2 molar ratio upon subtraction of the XD contribution (data not shown).

On the other hand, in CD experiments, inability of IRF-3 RD to interact with N_TAIL _could arise either from possible dimerization of IRF-3 RD in sodium phosphate buffer, with subsequent loss of binding ability, or from the use of protein concentrations below the actual K_D_. Indeed, while the N_TAIL _and XD concentrations (3.5 and 7 μM, respectively) are well above the reported K_D _(100 nM) [38], the K_D _between N_TAIL _and IRF-3 RD is not known. Hence, the experimentally used N_TAIL _and IRF-3 RD concentrations (1.4 and 2.8 μM, respectively) might be not high enough to allow a productive interaction.

In order to circumvent these problems, we carried out NMR experiments, which allowed both use of buffer D, a condition where IRF-3 RD was shown to be monomeric, and of protein concentrations as high as 100 μM. Under these conditions, no interaction was detected between IRF-3 RD and either N_TAIL _or the full-length nucleoprotein. Lack of interaction with N ruled out the possibility that IRF-3 RD might interact with N_TAIL _only in the context of a self-assembled nucleocapsid-like structure. Noteworthy, using comparable protein concentrations, heteronuclear NMR has already been proven to be sensitive enough to document the XD-induced folding of N_TAIL_, where from a total of 125 residues, 11 were shown to undergo an α-helical transition and seven a less dramatic conformational change [38]. The N_TAIL_-XD interaction is however characterized by a high affinity, with an estimated K_D _of 100 nM [38]. We could speculate that if the N_TAIL_-IRF-3 RD binding affinity is much weaker, then this would result in little complex formation, thus escaping detection. However, it should be pointed out that heteronuclear NMR has been already proven to be sensitive enough to document protein-protein interactions characterized by K_D _values up to the mM range (for a review see [46]). Notably, using protein concentrations similar to those used in this study, heteronuclear NMR successfully unveiled the weak-affinity interaction (K_D _10 μM) between the intrinsically disorder cyclin-dependent inhibitor p21 and Cdk2 [47].

Furthermore, if we assume that in HSQC experiments the percentage of the N_TAIL_/IRF-3 RD complex is as low as 10%, which could indeed escape detection, then the corresponding calculated K_D _would be approximately 0.9 mM, based on the following equation

(1)

A K_D _in the mM range would support a fortuitous interaction at best, assuming an IRF-3 intracellular concentration of approximately 1 μM, as calculated assuming an overall intracellular protein concentration of 200 mg/ml and that IRF-3 represents approximately 1/4000 of total cellular proteins [48].

Using SDSL EPR spectroscopy and a monomeric form of IRF-3 RD, we failed to point out an impact of this latter on the mobility of three spin labels grafted within N_TAIL_. These results support a lack of involvement of the three spin-labeled sites in the interaction and/or a lack of interaction between N_TAIL _and IRF-3 RD. Although this former hypothesis could not be formally ruled out, the spin labels are located within three N_TAIL _regions that can be expected to be involved in the possible interaction with IRF-3 RD, since they are conserved within *Morbillivirus *members [49] and have been shown to play a functional role in the molecular partnership of N_TAIL_: indeed, Box1 is involved in the interaction with a yet unidentified nucleoprotein cellular receptor [22,23], while Box2 and Box3 participate to binding to both Hsp72 [20,21] and XD [28,29,38]. Besides, it is worthy to mention that spin-label EPR spectroscopy has already been proven to be well suited to monitor low-affinity interactions, such as binding of spin-labeled ATP to the multi-drug resistance P-glycoprotein that is characterized by a K_D _of approximately 700 μM [50].

Lack of N_TAIL _impact on the intrinsic trp fluorescence of IRF-3 RD could reflect either a lack of interaction between the proteins, or a location of the IRF-3 RD trp residues outside the region of interaction. This latter hypothesis is however unlikely, since the 9 trp residues are scattered on the whole IRF-3 RD surface (see Figure 1B), with two of them being located in the proximity of the triple α-helical bundle that is supposed to correspond to the putative binding site, based on its similarity to the XD structure (see pdb file 1QWT) and on its involvement in the binding of the (otherwise disordered) IBiD domain of CREB (see pdb file 1ZOQ). Taking into account the fact that fluorescence spectroscopy has been already successfully used to monitor the interaction between XD and a single-site tryptophan-substituted N_TAIL _variant [38] and has also been reported to be able to unveil weak affinity interactions with a K_D _in the 20–30 μM range [51], these data argue for a lack of direct interaction between IRF-3 RD and N_TAIL_.

Since co-immunoprecipitation experiments carried out by both tenOever *et al*. [24] and ourselves, suggest that N and IRF-3 interact somehow, the hypothesis can be drawn that a specific cellular context is required for the interaction to occur. We therefore questioned whether a bacterial lysate could provide such a context. The interaction was thus tested in crude *E. coli *lysates using a co-immunoprecipitation protocol in which no washing step was carried out, a method derived from the "hold-up" technique that is well adapted for the detection of low-affinity interactions with K_D _values as high as 50 μM [52]. Notably, and in spite of the fact that the experimental design was in principle expected to allow documentation of low-affinity and kinetically transient complexes, no interaction could be detected. Likewise, using the yeast two-hybrid assay, an approach that has been already successfully used to document MeV protein-protein interactions [42], no interaction was detected in the yeast cellular context either.

## Conclusion

Altogether, the results herein presented indicate that the N_TAIL_-IRF-3 interaction requires a specific eukaryotic cellular environment, such as that provided by 293T cells. That a specific cellular context is required for efficient MeV RNA synthesis has been already reported [53] and argue for the requirement of unknown cellular co-factor(s) in conferring competence for both transcription and replication to viral nucleocapsids. In the case of the N_TAIL_-IRF-3 interaction, the strict dependence from a particular cellular context may reflect the requirement of either a human- or mammalian-specific post-translational modification of one or both interactors, or of a human or mammalian cellular co-factor, which would act as a bridge thereby promoting the N-IRF-3 association. In support of this last hypothesis, intrinsically disordered proteins are known to often display weak affinities towards their partners [7,15], thus leading to complexes that are not stable by themselves and must be strengthened by the combination of other interactions or by multimerization (for examples within the replicative complex of MeV see [54]). In further support of the requirement for a cellular co-factor, tenOever et al. found that the N protein associated with both IRF-3 and the IRF-3 virus-activated kinase suggesting that both proteins are part of a large complex that favors the colocalization of the kinase and of its substrate [24]. In addition, as MeV infection (or MeV N transfection) triggers binding of IRF-3 to the CREB binding protein to form a complex that activates target genes in the nucleus [24,55], it is also possible that recognition of N by IRF-3 could be promoted by the CREB binding protein.

Preparative co-immunoprecipitation experiments coupled to mass spectrometry are in progress in view of either ascertaining a role for the virus-activated kinase or the CREB binding protein, or identifying a possible cellular co-factor distinct from these two latter cellular proteins.

## Competing interests

The authors declare that they have no competing interests.

## Authors' contributions

MCol expressed and purified both unlabeled and ^15^N-labeled IRF-3 RD and searched for buffer conditions leading to a monomeric form. He also purified XD and both unlabeled and ^15^N-labeled N_TAIL_, performed co-immunoprecipitation and co-precipitation experiments from bacterial lysates, carried out CD and fluorescence studies and prepared samples for NMR and EPR studies. JMB cloned IRF-3 RD and carried out preliminary interaction studies with a dimeric form of IRF-3 RD. CC carried out yeast two-hybrid experiments. CS cloned IRF-3 cDNA by RT-PCR. SV carried out co-immunoprecipitation experiments in human cells. SC purified and spin-labeled N_TAIL _cys variants. VB and AF recorded the EPR spectra, while HD recorded the NMR spectra. MCou participated to co-immunoprecipitation and co-precipitation experiments from bacterial lysates. SL and DG are both responsible for the study design and coordination of the work, with SL being in charge of molecular biology, biochemistry and biophysics aspects and DG being in charge of cellular biology aspects. The manuscript was written by SL with an important contribution by DG. All authors read and approved the final manuscript.

## References

[B1] Lamb RA, Kolakofsky D, Fields BN, Knipe DM, Howley PM (2001). *Paramyxoviridae*: The Viruses and Their Replication. "Fields Virology".

[B2] Karlin D, Longhi S, Canard B (2002). Substitution of two residues in the measles virus nucleoprotein results in an impaired self-association. Virology.

[B3] Kingston RL, Walter AB, Gay LS (2004). Characterization of nucleocapsid binding by the measles and the mumps virus phosphoprotein. J Virol.

[B4] Longhi S, Receveur-Brechot V, Karlin D, Johansson K, Darbon H, Bhella D, Yeo R, Finet S, Canard B (2003). The C-terminal domain of the measles virus nucleoprotein is intrinsically disordered and folds upon binding to the C-terminal moiety of the phosphoprotein. J Biol Chem.

[B5] Heggeness MH, Scheid A, Choppin PW (1980). Conformation of the helical nucleocapsids of paramyxoviruses and vesicular stomatitis virus: reversible coiling and uncoiling induced by changes in salt concentration. Proc Natl Acad Sci USA.

[B6] Heggeness MH, Scheid A, Choppin PW (1981). The relationship of conformational changes in the Sendai virus nucleocapsid to proteolytic cleavage of the NP polypeptide. Virology.

[B7] Wright PE, Dyson HJ (1999). Intrinsically unstructured proteins: re-assessing the protein structure-function paradigm. J Mol Biol.

[B8] Uversky VN, Gillespie JR, Fink AL (2000). Why are "natively unfolded" proteins unstructured under physiologic conditions?. Proteins.

[B9] Dunker AK, Lawson JD, Brown CJ, Williams RM, Romero P, Oh JS, Oldfield CJ, Campen AM, Ratliff CM, Hipps KW, Ausio J, Nissen MS, Reeves R, Kang C, Kissinger CR, Bailey RW, Griswold MD, Chiu W, Garner EC, Obradovic Zl (2001). Intrinsically disordered protein. J Mol Graph Model.

[B10] Dunker AK, Obradovic Z (2001). The protein trinity – linking function and disorder. Nat Biotechnol.

[B11] Tompa P (2002). Intrinsically unstructured proteins. Trends Biochem Sci.

[B12] Uversky VN (2002). Natively unfolded proteins: a point where biology waits for physics. Protein Sci.

[B13] Tompa P (2003). The functional benefits of disorder. J Mol Structure (Theochem).

[B14] Fink AL (2005). Natively unfolded proteins. Curr Opin Struct Biol.

[B15] Dyson HJ, Wright PE (2005). Intrinsically unstructured proteins and their functions. Nat Rev Mol Cell Biol.

[B16] Uversky VN, Oldfield CJ, Dunker AK (2005). Showing your ID: intrinsic disorder as an ID for recognition, regulation and cell signaling. J Mol Recognit.

[B17] Radivojac P, Iakoucheva LM, Oldfield CJ, Obradovic Z, Uversky VN, Dunker AK (2007). Intrinsic disorder and functional proteomics. Biophys J.

[B18] Dunker AK, Oldfield CJ, Meng J, Romero P, Yang JY, Chen JW, Vacic V, Obradovic Z, Uversky VN (2008). The unfoldomics decade: an update on intrinsically disordered proteins. BMC Genomics.

[B19] Dunker AK, Silman I, Uversky VN, Sussman JL (2008). Function and structure of inherently disordered proteins. Curr Opin Struct Biol.

[B20] Zhang X, Glendening C, Linke H, Parks CL, Brooks C, Udem SA, Oglesbee M (2002). Identification and characterization of a regulatory domain on the carboxyl terminus of the measles virus nucleocapsid protein. J Virol.

[B21] Zhang X, Bourhis JM, Longhi S, Carsillo T, Buccellato M, Morin B, Canard B, Oglesbee M (2005). Hsp72 recognizes a P binding motif in the measles virus N protein C-terminus. Virology.

[B22] Laine D, Trescol-Biémont M, Longhi S, Libeau G, Marie J, Vidalain P, Azocar O, Diallo A, Canard B, Rabourdin-Combe C, Valentin H (2003). Measles virus nucleoprotein binds to a novel cell surface receptor distinct from FcgRII via its C-terminal domain: role in MV-induced immunosuppression. J Virol.

[B23] Laine D, Bourhis J, Longhi S, Flacher M, Cassard L, Canard B, Sautès-Fridman C, Rabourdin-Combe C, Valentin H (2005). Measles virus nucleoprotein induces cell proliferation arrest and apoptosis through NTAIL/NR and NCORE/FcgRIIB1 interactions, respectively. J Gen Virol.

[B24] tenOever BR, Servant MJ, Grandvaux N, Lin R, Hiscott J (2002). Recognition of the Measles Virus Nucleocapsid as a Mechanism of IRF-3 Activation. J Virol.

[B25] Hiscott J (2007). Triggering the innate antiviral response through IRF-3 activation. J Biol Chem.

[B26] Johansson K, Bourhis JM, Campanacci V, Cambillau C, Canard B, Longhi S (2003). Crystal structure of the measles virus phosphoprotein domain responsible for the induced folding of the C-terminal domain of the nucleoprotein. J Biol Chem.

[B27] Oldfield CJ, Cheng Y, Cortese MS, Romero P, Uversky VN, Dunker AK (2005). Coupled Folding and Binding with alpha-Helix-Forming Molecular Recognition Elements. Biochemistry.

[B28] Bourhis J, Johansson K, Receveur-Bréchot V, Oldfield CJ, Dunker AK, Canard B, Longhi S (2004). The C-terminal domain of measles virus nucleoprotein belongs to the classof intrinsically disordered proteins that fold upon binding to their pohysiological partner. Virus Research.

[B29] Morin B, Bourhis JM, Belle V, Woudstra M, Carrière F, BGuigliarelli B, Fournel A, Longhi S (2006). Assessing induced folding of an intrinsically disordered protein by site-directed spin-labeling EPR spectroscopy. J Phys Chem B.

[B30] Belle V, Rouger S, Costanzo S, Liquiere E, Strancar J, Guigliarelli B, Fournel A, Longhi S (2008). Mapping alpha-helical induced folding within the intrinsically disordered C-terminal domain of the measles virus nucleoprotein by site-directed spin-labeling EPR spectroscopy. Proteins.

[B31] Kingston RL, Hamel DJ, Gay LS, Dahlquist FW, Matthews BW (2004). Structural basis for the attachment of a paramyxoviral polymerase to its template. Proc Natl Acad Sci USA.

[B32] Qin BY, Liu C, Lam SS, Srinath H, Delston R, Correia JJ, Derynck R, Lin K (2003). Crystal structure of IRF-3 reveals mechanism of autoinhibition and virus-induced phosphoactivation. Nat Struct Biol.

[B33] Qin BY, Liu C, Srinath H, Lam SS, Correia JJ, Derynck R, Lin K (2005). Crystal structure of IRF-3 in complex with CBP. Structure.

[B34] Demarest SJ, Deechongkit S, Dyson HJ, Evans RM, Wright PE (2004). Packing, specificity, and mutability at the binding interface between the p160 coactivator and CREB-binding protein. Protein Sci.

[B35] Chen M, Cortay JC, Logan IR, Sapountzi V, Robson CN, Gerlier D (2005). Inhibition of ubiquitination and stabilization of human ubiquitin E3 ligase PIRH2 by measles virus phosphoprotein. J Virol.

[B36] Plumet S, Gerlier D (2005). Optimized SYBR green real-time PCR assay to quantify the absolute copy number of measles virus RNAs using gene specific primers. J Virol Methods.

[B37] Brizzard BL, Chubet RG, Vizard DL (1994). Immunoaffinity purification of FLAG epitope-tagged bacterial alkaline phosphatase using a novel monoclonal antibody and peptide elution. Biotechniques.

[B38] Bourhis JM, Receveur-Bréchot V, Oglesbee M, Zhang X, Buccellato M, Darbon H, Canard B, Finet S, Longhi S (2005). The intrinsically disordered C-terminal domain of the measles virus nucleoprotein interacts with the C-terminal domain of the phosphoprotein via two distinct sites and remains predominantly unfolded. Protein Sci.

[B39] Uversky VN (1993). Use of fast protein size-exclusion liquid chromatography to study the unfolding of proteins which denature through the molten globule. Biochemistry.

[B40] Mori S, Abeygunawardana C, Johnson MO, van Zijl PC (1995). Improved sensitivity of HSQC spectra of exchanging protons at short interscan delays using a new fast HSQC (FHSQC) detection scheme that avoids water saturation. J Magn Reson B.

[B41] Piotto M, Saudek V, Sklenar V (1992). Gradient-tailored excitation for single-quantum NMR spectroscopy of aqueous solutions. J Biomol NMR.

[B42] Chen M, Cortay JC, Gerlier D (2003). Measles virus protein interactions in yeast: new findings and caveats. Virus Res.

[B43] Feix JB, Klug CS (1998). Site-directed spin-labeling of membrane proteins and peptide-membrane interactions. Biological magnetic resonance Volume Spin labeling: the next millenium.

[B44] Hubbell WL, Gross A, Langen R, Lietzow MA (1998). Recent advances in site-directed spin labeling of proteins. Curr Opin Struct Biol.

[B45] Biswas R, Kuhne H, Brudvig GW, Gopalan V (2001). Use of EPR spectroscopy to study macromolecular structure and function. Sci Prog.

[B46] Shi Y, Wu J (2007). Structural basis of protein-protein interaction studied by NMR. J Struct Funct Genomics.

[B47] Kriwacki RW, Hengst L, Tennant L, Reed SI, Wright PE (1996). Structural studies of p21Waf1/Cip1/Sdi1 in the free and Cdk2-bound state: conformational disorder mediates binding diversity. Proc Natl Acad Sci USA.

[B48] Luby-Phelps K (2000). Cytoarchitecture and physical properties of cytoplasm: volume, viscosity, diffusion, intracellular surface area. Int Rev Cytol.

[B49] Diallo A, Barrett T, Barbron M, Meyer G, Lefevre PC (1994). Cloning of the nucleocapsid protein gene of peste-des-petits-ruminants virus: relationship to other morbilliviruses. J Gen Virol.

[B50] Delannoy S, Urbatsch IL, Tombline G, Senior AE, Vogel PD (2005). Nucleotide binding to the multidrug resistance P-glycoprotein as studied by ESR spectroscopy. Biochemistry.

[B51] Murakami K, Andree PJ, Berliner LJ (1982). Metal ion binding to alpha-lactalbumin species. Biochemistry.

[B52] Charbonnier S, Zanier K, Masson M, Trave G (2006). Capturing protein-protein complexes at equilibrium: the holdup comparative chromatographic retention assay. Protein Expr Purif.

[B53] Vincent S, Tigaud I, Schneider H, Buchholz CJ, Yanagi Y, Gerlier D (2002). Restriction of measles virus RNA synthesis by a mouse host cell line: trans-complementation by polymerase components or a human cellular factor(s). J Virol.

[B54] Bourhis JM, Canard B, Longhi S (2006). Structural disorder within the replicative complex of measles virus: functional implications. Virology.

[B55] Chen W, Srinath H, Lam SS, Schiffer CA, Royer WE, Lin K (2008). Contribution of Ser386 and Ser396 to activation of interferon regulatory factor 3. J Mol Biol.

